# The Role of Sirtuins in Osteogenic Differentiation of Vascular Smooth Muscle Cells and Vascular Calcification

**DOI:** 10.3389/fcvm.2022.894692

**Published:** 2022-06-01

**Authors:** Shuangshuang Wang, Siwang Hu

**Affiliations:** ^1^Department of Cardiology, The First People's Hospital of Wenling (The Affiliated Wenling Hospital of Wenzhou Medical University), Wenling, China; ^2^The Orthopedic Center, The First People's Hospital of Wenling (The Affiliated Wenling Hospital of Wenzhou Medical University), Wenling, China

**Keywords:** sirtuins, vascular calcification, therapy, VSMCs, osteogenic differentiation

## Abstract

Vascular calcification (VC) is a common pathological change in many chronic diseases, such as diabetes and chronic kidney disease. It is mainly deposited in the intima and media of vessels in the form of hydroxyapatite. Recently, a lot of research has been performed to show that VC is associated with various cellular stresses, such as hyperphosphate, hyperglycemia and oxidative stress. Unfortunately, our understanding of the pathogenesis of calcification is far from comprehensive. Sirtuins belong to a family of class III highly conserved deacetylases that are involved in the regulation of biological and cellular processes including mitochondrial biogenesis, metabolism, oxidative stress, inflammatory response, DNA repair, etc. Numerous studies have shown that sirtuins might play protective roles in VC, and restoring the activity of sirtuins may be a potentially effective treatment for VC. However, the exact mechanism of their vascular protection remains unclear. Here, we reviewed the roles of sirtuins in the osteogenic transformation of vascular smooth muscle cells and the development of VC. We also elucidated the applications of sirtuins agonists for the treatment of VC.

## Introduction

As the aging trend of the world population continues, vascular calcification (VC) is becoming a major global health problem. VC is characterized by the deposition of hydroxyapatite in the vessel wall, which is mainly divided into intima calcification and medial calcification (1, 2). The former is common in patients with atherosclerosis, while medial calcification often occurs in patients with chronic kidney disease (CKD), diabetes, and hypertension, which causes arterial remodeling and vascular stiffness (3). The recent study showed that abdominal aortic calcification was associated with increased risk of atherosclerotic vascular disease mortality and all-cause mortality (4). And the patients with macrocalcifications had a three- to four-fold risk of developing a fatal cardiovascular event (5). Unfortunately, due to the unclear mechanism of VC, there is no effective drug or method for preventing or reversing VC. Therefore, it is urgent to deeply explore the pathogenesis of VC and develop new strategies to alleviate the onset and progression of VC.

Sirtuins (SIRTs) comprise a family of highly conserved nicotinamide adenine dinucleotide (NAD^+^)-dependent deacetylases, with seven homologous analogs in mammals, SIRT1-SIRT7 (6). The SIRT1-7 share a conserved 275-amino-acid catalytic core domain, but have different subcellular localization, among which SIRT1, SIRT6 and SIRT7 are mainly located in the nucleus, SIRT3, SIRT4 and SIRT5 in the mitochondria, and SIRT2 in the cytoplasm (7). SIRTs have been proven to be involved in the regulation of several pathophysiological processes via deacetylating various substrates, including cell metabolism, inflammation, oxidative stress, and aging (8–11). All these pathophysiological processes play crucial parts in VC. However, the mechanisms of different SIRTs homologs in VC are complicated, and the pathways vary from the homologs on account of their different localization in cells. In this review, we were dedicated to clarifying the role and exact mechanism of SIRTs during the occurrence and development of VC.

## The Pathophysiology of Vascular Calcification

VC is a significant feature of vascular changes in various physiological or pathological states. Increasing evidence shows that aging is an important contributor to VC (12). In addition to aging, many other risk factors including CKD, diabetes, hypertension, and dyslipidemia, might participate in the progression of VC (13). Notably, CKD can significantly increase the risk of cardiovascular disease, especially VC (14). The patients with CKD are frequently exposed to various pro-calcification factors, such as phosphate, indolyl sulfate, advanced glycation end products, and pro-inflammatory cytokines (15, 16). High phosphorus due to mineral imbalance in CKD drives the occurrence of VC, and protein-bound uremic toxin could further aggravate VC (17, 18). Besides, studies revealed that postmenopausal women were also at a significantly increased risk of VC (19–21). In recently menopausal women, estrogen supplementation could slow the progression of coronary artery calcification (22). Originally, VC was thought to be a passive degenerative process that marked the aging of blood vessels. However, more and more studies have shown that VC is an active process regulated by multiple factors, including abnormal calcium and phosphate homeostasis, oxidative stress, inflammation, matrix remodeling, and so on (23). Therefore, VC is a common disease with complex regulatory mechanisms.

The vessel wall is composed of endothelial cells, vascular smooth muscle cells (VSMCs), fibroblasts, and pericytes (24). Normally, a monolayer of endothelial cells forms the innermost layer that acts as a barrier between the vessel lumen and the vessel wall in order to maintain the non-thrombotic surface and quiescence in the vascular wall. Under pathological conditions, endothelial cells also respond to different stimuli and exist in various activation states, including inflammatory, angiogenic, and osteogenic phenotypes (25–27). Growing evidence suggests that endothelial cells influence the development of VC in a variety of pathways, including transition to mesenchymal and osteoblastic lineages, secretion of calcific growth factors, disruption of the proteolytic activity of IELs, induction of endothelial alkaline phosphatase and inappropriate interactions with underlying cells (27).

**Figure 1:**
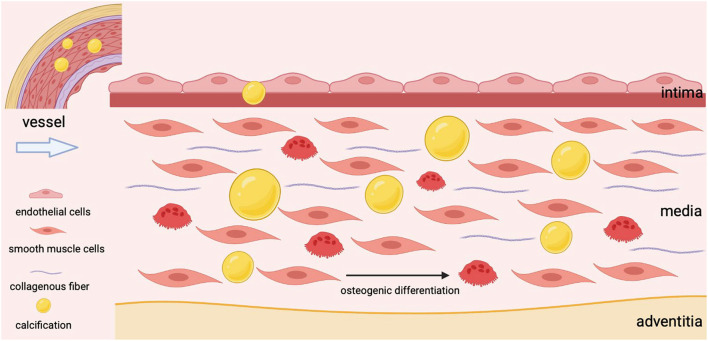
The osteogenic differentiation of VSMCs during vascular calcification.

As the most abundant cell type, VSMCs are mainly located in the tunica media with a high degree of plasticity and regulate the dilation and contraction of vessels (28). The senescence of VSMCs, the osteogenic transformation of VSMCs, the apoptosis of VSMCs, and the deposition of extracellular matrix have been confirmed as the reasons for VSMCs dysfunction and the development of VC (11). The transition of VSMCs from a contractile state to a secretory phenotype has been identified as a key event in VC, and the synthesized VSMCs undergoes further maladaptive osteogenic differentiation under continuous stimulation, ultimately leading to hydroxyapatite deposition in blood vessels (Figure 1) (29, 30). As VSMCs transition to an osteogenic phenotype upon specific stimuli, these cells could acquire the characteristics of osteoblasts (31). It is mainly manifested by increased expression of osteogenic markers such as ALP, BMP2, and RUNX2, while decreased expression of calcification-inhibitor protein (32). What's more, senescent VSMCs could also promote their osteogenic transformation (33). To sum up, VSMCs are involved in the process of VC from multiple perspectives.

## Sirtuins and Vascular Calcification

As classical deacetylases, SIRTs catalyze the deacetylation of proteins mainly by breaking the bond between NAD^+^ and nicotinamide ribosomes, and then participate in the regulation of various processes (34, 35). For example, SIRT1 might mediate cellular senescence by regulating p53 and p65 acetylation (33). And the decreased vascular SIRT1 expression was associated with age-related arteriosclerosis (36). In recent years, the molecular mechanism of SIRTs in the phenotypic changes of VSMCs has been extensively studied in VC. In the following, we mainly focused on the roles of SIRTs in VSMCs osteogenic differentiation and their potential as therapeutic targets for VC.

### SIRT1 and Vascular Calcification

SIRT1 is the well-studied family member of NAD^+^-dependent deacetylase, which could protect against cardiovascular diseases (37, 38). In Japanese hemodialysis patients, SIRT1 polymorphisms (rs7069102 and rs2273773) were confirmed to be associated with coronary artery calcification (39). *In-vitro* cultured aortas from SIRT1^−/−^ mice exhibited accelerated medial calcification upon phosphate stimulation (40). A transgenic mouse model was used to demonstrate that lifetime overexpression of SIRT1 might ameliorate aortic stiffness and prevent aortic calcification with advancing age (41). Therefore, SIRT1 plays an essential role in VC.

#### The Molecular Mechanism of SIRT1 in VC

**Figure 2:**
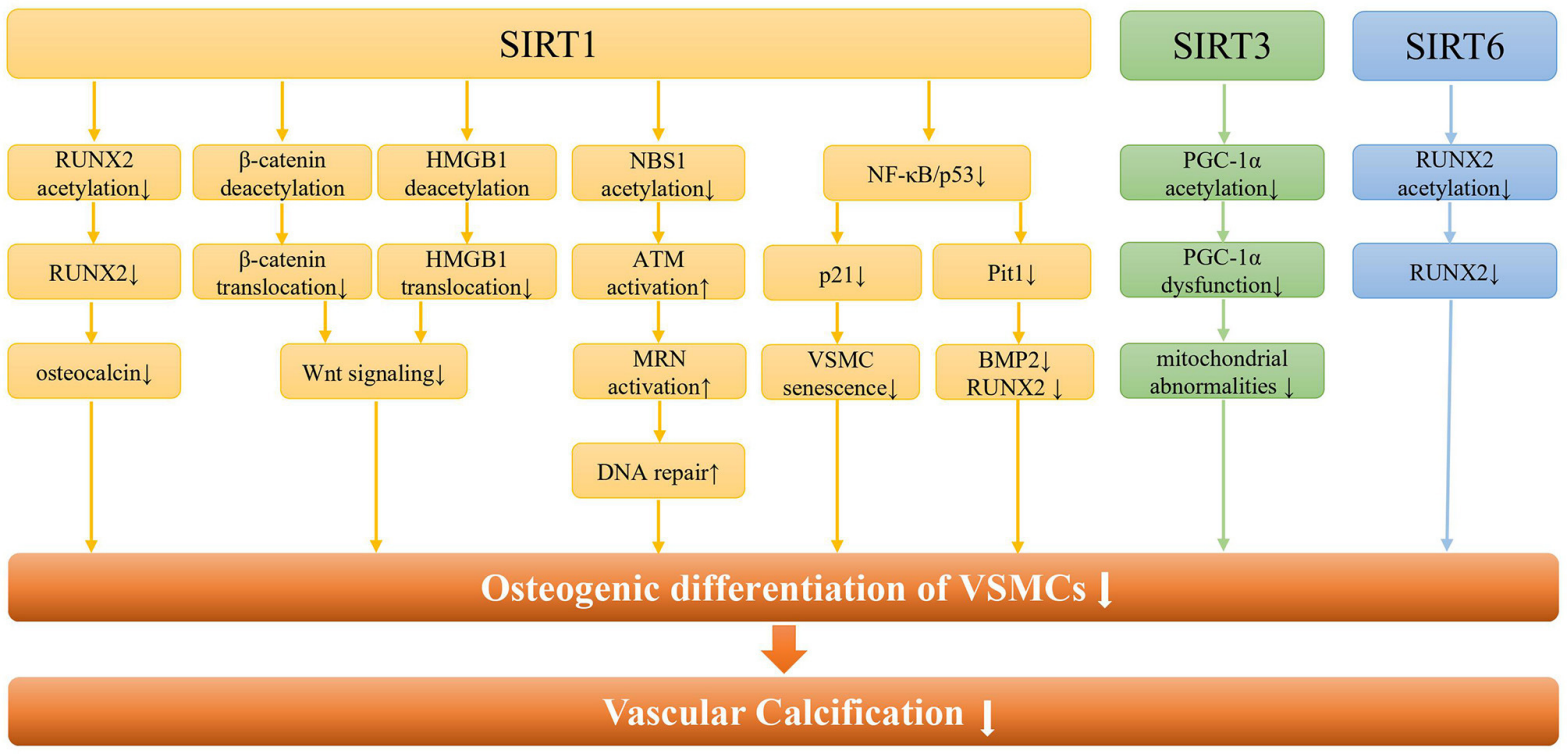
The molecular pathways of SIRTs in vascular calcification. Regarding SIRT1, it can regulate the osteogenic differentiation of VSMCs and vascular calcification through the RUNX2/osteocalcin pathway, the Wnt signaling pathway, the DNA repair pathway, and the NF-κB pathway. In addition, SIRT1 can alleviate the osteogenic differentiation of VSMCs by regulating the senescence of VSMCs through NF-κB/p53/p21 pathway. As for SIRT3, it is mainly involved in the osteogenic differentiation of VSMCs by regulating mitochondrial function through PGC-1a. Besides, SIRT6 regulates vascular calcification through RUNX2 deacetylation in CKD.

Existing evidence from cell and animal models indicated that SIRT1 was involved in VC through a variety of signaling pathways (Figure 2). Most importantly, SIRT1 could prevent arterial calcification via inhibition of VSMCs osteogenic differentiation (42). And SIRT1 could serve as a positive regulator of the osteoblast transcription factor, RUNX2 (43). Under high glucose conditions, the downregulation of SIRT1 expression promoted acetylation of the RUNX2 promoter region, thereby increasing the osteogenic differentiation of VSMCs (44). On the other hand, low expression of SIRT1 fails to deacetylate β-Catenin and HMGB1, thus promoting their translocation to the nucleus, and promoting the development of VC by activating Wnt signaling (45). The lncRNA HOTAIR can upregulate SIRT1 expression via miR-126/Klotho axis, and then attenuate VSMCs calcification and VC by inhibiting Wnt/β-catenin pathway (46). Additionally, SIRT1 downregulation may promote NBS1 acetylation and inhibit ATM activation, thereby inhibiting MRN complex formation, and ultimately leading to failure of DNA repair (47). And increased SIRT1 could promote MRN activation, leading to increased DNA repair and cell survival, ultimately attenuating DNA damage-induced VSMCs osteogenic differentiation and VC in diabetes (48).

Furthermore, phosphate-induced VSMCs calcification may be associated with both premature and replicative cellular senescence (42). With increasing age, the SIRT1 expression in human VSMCs was reduced, which led to reducing migratory and proliferative capacity, as well as induction of cellular senescence (49). Han et al. reported that upregulated SIRT1 expression could retard IL-1β-induced VSMCs senescence via NF-κB/p53/p21 pathways (50). In addition, high glucose and high phosphate can synergistically inhibit SIRT1 expression, increase p65 acetylation, and activate NF-κB signaling pathway, thereby increasing osteogenic transformation, cellular senescence, and ROS levels in VSMCs, resulting in VC (51). The upregulated miR-34a could promote senescence in VSMCs and ultimately trigger VC occurrence and progression (52, 53). And miR-34a directly downregulated Axl and SIRT1 to induce cellular senescence and promote VSMC mineralization and VC (54). Therefore, cell senescence might synergize with downregulation of SIRT1 to cause the osteogenic transformation of VSMCs, which ultimately leads to the occurrence of calcification.

All the above highlights that SIRT1 serves as a key factor in both senescence and osteogenic differentiation of VSMCs, which exist a synergistic role in VC. It provides new insights and rationale for future development of specific therapeutic targets with potent SIRT1 analogs.

#### SIRT1 as a Potential Therapeutic Target for VC

Based on the above evidence, SIRT1 is an important target in preventing the occurrence and development of VC. Strategies to enhance the activity of SIRT1 may be an effective mean to prevent and improve VC. In this regard, several SIRT1 activators have been synthesized to activate SIRT1, such as SRT1720, SRT2014 and SRT1460 (11). SRT1720 could protect VSMCs from calcification by activating SIRT1 in either osteogenesis or hyperglycemia condition (44, 51). Besides, some natural drugs have been shown to act as activators of SIRT1, including resveratrol, spermidine, terpene-4-ol, etc. (Table 1).

**Table 1:** The applications of SIRT1 activators in vascular calcification.

**SIRT1 activators**	**Backgrounds**	**Study Type**	**Effects**	**References**
SRT1720	Diabetes	*In-vitro* cell model	It can protect cells from calcification.	(44)
SRT1720	High phosphate and high glucose	*In-vitro* cell model	It can inhibit VSMCs senescence and osteogenic differentiation by inhibiting NF-κB activity, thereby inhibiting VC.	(51)
Resveratrol	Chronic kidney disease	*In-vitro* cell model	It can protect against vascular calcification via anti-oxidative stress.	(55)
Resveratrol	Menopause	In ovariectomized rats model	It can activate SIRT1 and regulate vascular calcification via OPG/RANKL pathway.	(56)
Terpene-4-ol	Chronic kidney disease	*In-vitro* cell model and *In-vivo* mice model	It can inhibit vascular calcification by activating SIRT1 and inhibiting PERK-eIF2 α-ATF4 signaling.	(57)
Spermidine	Chronic kidney disease	*In-vitro* cell model and *In-vivo* CKD rats model	It can protect VC in CKD by modulating SIRT1 and endoplasmic reticulum stress signaling.	(33)
Intermedin	Chronic kidney disease	*In-vitro* cell model and *In-vivo* CKD rats model	It can inhibit VC by increasing matrix gamma-carboxyglutamic acid protein levels.	(58)
Intermedin1-53	Chronic kidney disease	*In-vitro* cell model and *In-vivo* CKD rats model	It can attenuate VC in CKD by upregulation of α-Klotho.	(59)
Intermedin1-53	Aging	*In-vitro* cell model and *In-vivo* rats model	It can attenuate vascular calcification by upregulating SIRT1.	(60)

Resveratrol (3,5,4'-trihydroxy-trans-stilbene), known as phytoestrogens in grapes, peanuts, and legumes, is a scavenger for free radicals (61). It has been reported that resveratrol could inhibit the oxidative stress-induced proliferation of VSMCs by activating SIRT1 signaling pathway, thereby improving cardiovascular diseases (61). Zhang et al. found that resveratrol could effectively improve VC in patients with end-stage renal disease, and the mechanism may be to inhibit the oxidative damage of VSMCs by regulating SIRT1 and Nrf2 (55). On the other hand, since resveratrol has a similar structure to estrogen, it can bind to estrogen receptors to produce similar effects. Resveratrol could reduce the expression levels of ALP, RUNX2 and increase the expression levels of OPG in the aortas of ovariectomized rats (56). In addition, the authors speculated that resveratrol might play key roles in regulating aortic calcification through SIRT1 activation. Therefore, resveratrol may be a potential drug for VC.

Terpene-4-ol is a common component in plant essential oils, which has anti-inflammatory and anti-tumor effects (62, 63). It has been reported to reduce calcium deposition in VSMCs and CKD mouse arteries and inhibit the phenotype switching of VSMCs (57). Mechanistically, terpinen-4-ol exerts anti-calcification effects by activating SIRT1 to deacetylate PERK and then inhibiting the endoplasmic reticulum stress pathway–PERK-eIF2α-ATF4 signaling pathway. Moreover, endoplasmic reticulum stress increases the expression of ATF4, which could bind to the RUNX2 promoter, affecting VSMC calcification (64, 65). Taken together, terpinen-4-ol could be a promising therapeutic agent for CKD-related VC.

The natural polyamine spermidine, a novel autophagy inducer and longevity elixir, could extend lifespan and reduce oxidative stress (66, 67). The previous study showed that spermidine exerted an anti-aging effect to reduce arterial sclerosis by increasing NO bioavailability, reducing oxidative stress, modifying structural factors, and enhancing autophagy (68). In addition, spermidine could improve cardiomyocyte aging by activating mitochondrial biogenesis and function through SIRT1-mediated deacetylation of peroxisome proliferator-activated receptor gamma coactivator-1α (PGC-1α) (69). Spermidine has been shown to protect VC in CKD by modulating SIRT1 and endoplasmic reticulum stress signaling (33). Moreover, spermidine was confirmed with the low toxicity and strong efficacy, suggesting it is a promising VC therapy in clinical application (70).

Intermedin (IMD) is a paracrine/endocrine peptide belonging to the calcitonin gene-related peptide superfamily, and IMD1-53 may be the main active fragment of IMD (71). IMD1-53 could improve vascular function by increasing endothelial nitric oxide synthase activity and inhibiting oxidative stress (72, 73). Studies have shown that IMD1-53 may attenuate VC in CKD by upregulating α-Klotho, and IMD could alleviate vitamin D3 plus nicotine (VDN)-induced VC by increasing the level of matrix gamma-carboxyglutamic acid protein (58, 59). Chen et al. found that IMD1-53 upregulated SIRT1 to protect senescence-associated VSMC calcification through PI3K/Akt, AMPK and cAMP/PKA signaling (60). In general, IMD1-53 has protective effects on VC caused by different causes through various pathways.

Overall, both natural and synthetic substances can relieve VC by improving SIRT1 activity. There is still a need to further explore efficient and safe drugs. Of course, diet and exercise also play an important role in vascular health. For example, calorie restriction can promote eNOS activity and SIRT1 expression, thereby reducing atherosclerosis development (74). Therefore, the therapeutic effects and mechanisms of diet and exercise in VC are worthy of study.

### SIRT3 and Vascular Calcification

As a mitochondrial sirtuin with the highest deacetylase activity, SIRT3 could regulate the acetylation levels of several mitochondrial proteins, thereby regulating mitochondrial energy metabolism, redox balance and mitochondrial dynamics (75). Mitochondria are the main energy source for maintaining cardiac function, and mitochondrial dysfunction regulated by SIRT3 can lead to the progression of various cardiac diseases, including hypertension, coronary atherosclerosis, and heart failure (76). Feng et al. found that SIRT3 expression was decreased in β GP-induced calcification of VSMCs and in arteries of CKD rats and patients (77). Downregulation of SIRT3 might induce an increase in the acetylation of PGC-1α, leading to PGC-1α dysfunction, which also compromised mitochondrial adenosine triphosphate (ATP) production and morphology damage, ultimately triggering VSMCs phenotypic transition and calcium deposition. Furthermore, deletion of soluble epoxide hydrolase was reported to stabilize and increase SIRT3, thereby ameliorating mitochondrial abnormalities and VC in CKD or high phosphate conditions (78). Currently, berberine, honokiol, and resveratrol have been confirmed to target SIRT3 to correct pathological condition. However, no drug targeting SIRT3 has been used in the treatment of VC, which should be investigated in the future.

### SIRT6 and Vascular Calcification

SIRT6, not only a chromatin-associated deacetylase but also a mono-ADP-ribosyltransferase, has been recognized to serve a pivotal role in a variety of biological processes, including DNA damage repair, cellular metabolism, etc (79). Previous studies showed that the downregulation of SIRT6 was involved in kidney injury, atherosclerosis, and cholesterol accumulation (79, 80). SIRT6 can attenuate CKD fibrosis by blocking the expression of β-catenin target genes through deacetylation (81). In addition, decreased SIRT6 was associated with an increased risk of VC in CKD patients (82). And SIRT6 could inhibit VSMCs osteogenic differentiation and attenuate VC through deacetylating RUNX2 and promoting its ubiquitination and subsequent degradation. Moreover, bone marrow mesenchymal stem cell exosomes could inhibit hyperphosphate-induced aortic calcification via the SIRT6-HMGB1 deacetylation pathway (83). In short, SIRT6 acts as a protective regulator and a potential therapeutic target for VC.

## Discussion and Conclusion

Acetylation and deacetylation of proteins regulate a variety of cellular biological processes, including cell proliferation, gene transcription, apoptosis, protein stability, and mitochondrial metabolism, which are closely related to cancers and cardiovascular diseases (76, 84). Accumulating studies showed that the deacetylase family SIRTs were essential for regulating several aspects of VC. In this review, we mainly introduced the regulatory roles of SIRTs in the osteogenic differentiation of VSMCs. The downregulation of both SIRT1 and SIRT6 could promote RUNX2 acetylation, thereby enabling osteogenic transformation of VSMCs. Also, SIRT1 can alleviate VC by regulating Wnt signaling pathway and DNA repair pathway. And the mitochondrial sirtuin SIRT3 could maintain mitochondrial function and inhibit VC by regulating mitochondrial protein acetylation levels. It suggests that SIRTs may form complex regulatory networks through multiple pathways to participate in VC.

SIRT2, the major cytoplasmic deacetylase, is downregulated in aging as well as cardiovascular diseases, including cardiac hypertrophy and heart failure (85). Specially, SIRT2 can protect vascular endothelial cells by inhibiting oxidative stress and regulating apoptosis (86). SIRT4 and SIRT5 are two other mitochondrial sirtuins, which have been reported to be regulators of cellular metabolism (87). For instance, mitochondrial SIRT3, 4 and 5 may affect cell metabolism and ROS homeostasis, ultimately regulating cardiovascular health (88). Interestingly, on the one hand, SIRT4 could attenuate inflammatory damage to human umbilical vein endothelial cells (89, 90). On the other hand, SIRT4 might increase ROS levels under Ang II-induced pathological stimulation and promote hypertrophic growth, fibrosis, and cardiac dysfunction (91). Therefore, the role and mechanism of SIRT4 are complex and must be explored in different states. SIRT7 mainly resides in the nucleolus and is involved in the regulation of overall genome stability, aging, and cell metabolism (92). Current evidence suggests that the SIRTs play important regulatory roles in VC. In addition to SIRT1, SIRT3 and SIRT6 mentioned above, the roles of other SIRTs family members in VC should also be explored to gain a more objective understanding of the SIRTs family.

Recently, miR-34a and lncRNA HOTAIR /miR-126/Klotho axis have been recognized to serve as upstream pathways of SIRT1. And SIRT1 could be involved in the regulation of VC through several downstream pathways, including the RUNX2/osteocalcin pathway, Wnt signaling pathway, DNA repair pathway, and NF-κB pathway. Moreover, myocardin, a myogenic coactivator responsible for the VSMC contractile phenotype, could interact directly with SIRT1. And maintenance of myocardin and SIRT1 and the normal contractile phenotype of VSMCs may be a protective mechanism of VC (93, 94). Interestingly, SIRT6 is the deacetylation target of SIRT1, and the two could work together to enhance DNA damage repair (95). The potential interaction between sirtuins and other molecules in VC should be validated in the future.

Oxidative stress plays an important role in VC in both CKD and diabetes. The elevated reactive oxygen species production in VSMCs can increase the expression of RUNX2, which is involved in the osteogenic transition of VSMCs and the occurrence of VC (31, 96). Though different SIRTs are in different locations, evidence suggests that SIRT1, SIRT3, and SIRT6 can all be involved in the regulation of oxidative stress. And the SIRT1 agonists have antioxidant effects during the treatment of VC, including resveratrol, spermidine, terpene-4-ol, and IMD. All these highlight the important regulatory role of SIRT1 and oxidative stress in VC. It also provides a theoretical basis for future randomized controlled trial studies of SIRT1 agonists in VC, thereby improving the current situation where no drugs are available.

Most previous studies have shown that SIRT1 levels of patients with advanced coronary disease and diabetic kidney disease decreased compared with healthy subjects (97, 98). As for uremic patients undergoing hemodialysis, the concentration of SIRT1 increased and was positively correlated with dialysis time (93). Angelika et al. found that the serum SIRT1 concentration in the CKD group was higher than that in the control group, and serum levels of SIRT1 tend to increase with the progression of CKD (99). This may be inconsistent with our above-mentioned tone, and the reasons for this need to be further explored in the future.

In conclusion, SIRTs play key roles in vascular protection by regulating multiple signaling pathways. SIRTs might be potential therapeutic targets to inhibit VSMC osteogenic differentiation and alleviate VC.

## Author Contributions

SH contributes to the conception, design, and final approval of the submitted version. SW contributes to completing the table and writing the paper. Both authors contributed to the article and approved the submitted version.

## Funding

This research was supported by the grant from National Natural Science Foundation of China (81900441), Natural Science Foundation of Zhejiang Province (LQ19H020002), Zhejiang Provincial Program for Medicine and Health (2022KY446), Social Development Science and Technology Foundation of Taizhou (21ywb115, 21ywb118, and 20ywb143), and Social Development Science and Technology Foundation of Wenling (2021S00197, 2020S0180083, and 2021S00156).

## Conflict of Interest

The authors declare that the research was conducted in the absence of any commercial or financial relationships that could be construed as a potential conflict of interest.

## Publisher's Note

All claims expressed in this article are solely those of the authors and do not necessarily represent those of their affiliated organizations, or those of the publisher, the editors and the reviewers. Any product that may be evaluated in this article, or claim that may be made by its manufacturer, is not guaranteed or endorsed by the publisher.
